# Liquid fuel cells

**DOI:** 10.3762/bjnano.5.153

**Published:** 2014-08-29

**Authors:** Grigorii L Soloveichik

**Affiliations:** 1 General Electric Global Research, Niskayuna, NY 12309, USA

**Keywords:** anion exchange membranes, direct alcohol fuel cells, direct borohydride fuel cells, electrocatalysts, liquid fuel cells, organic fuel, proton exchange membranes

## Abstract

The advantages of liquid fuel cells (LFCs) over conventional hydrogen–oxygen fuel cells include a higher theoretical energy density and efficiency, a more convenient handling of the streams, and enhanced safety. This review focuses on the use of different types of organic fuels as an anode material for LFCs. An overview of the current state of the art and recent trends in the development of LFC and the challenges of their practical implementation are presented.

## Introduction

Fuel cells are considered to be one of the key elements of the “hydrogen economy”, in which hydrogen generated from renewable energy sources would be widely used as a clean energy carrier [1]. They do not produce greenhouse gases and other pollutants during their operation, and they have a higher efficiency entitlement (no Carnot cycle limitation) and lower maintenance (no moving parts) than internal combustion engines [2]. The total reaction of hydrogen oxidation in a fuel cell is described by Equation 1 and the cell has an open circuit potential (OCP) of 1.23 V under ambient conditions.

[1]
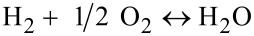


**Figure 1:**
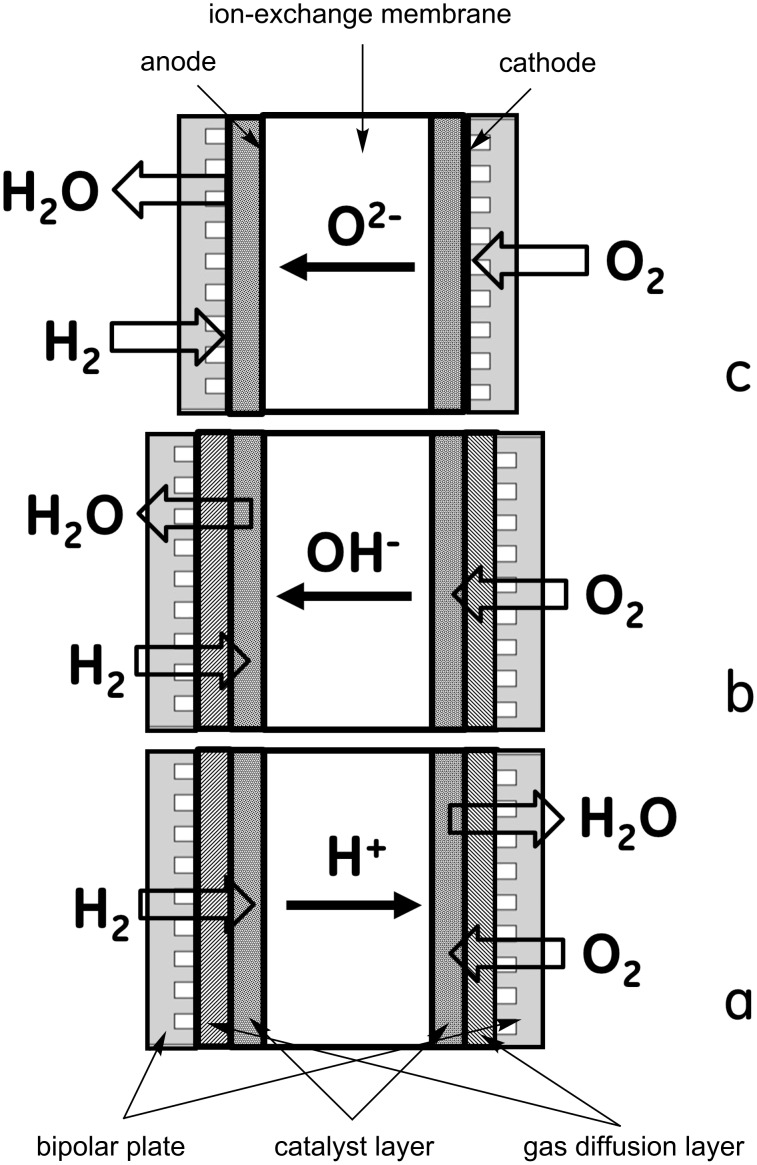
The electrochemistry and major components of liquid fuel cells with proton (a), alkaline (b) and solid oxide (c) ion-exchange membranes.

There are three major types of hydrogen/air fuel cells differing in the types of ions (protons, hydroxyl, and oxygen anions) transported through the membrane (Figure 1). In all cases the structure of the fuel cell is similar and consists of a cathode and an anode with a current collector (bipolar plate), a gas diffusion layer, and a catalyst layer. The electrodes are separated by an ion-conducting insulating membrane (Figure 1). Bipolar or field plates separating the individual cells in the stack should have a high corrosion resistance, good electronic and thermal conductivity, and be designed to evenly distribute reactants and products. It is worth noting that the bipolar plates have an impact on the cost structure comparable with the impact of catalytic electrodes [3].

In a solid oxide fuel cell (SOFC) the electrolyte conducting the negative oxygen ions (Figure 1c) is usually a rare-earth metal oxide doped zirconia, e.g., yttria stabilized zirconia (YSZ) or ceria that operates at high temperature (700–1000 °C). Liquid fuels may be used directly in SOFCs without reforming. For example, toluene, *n*-decane, and synthetic diesel fuel were fed to a SOFC at 700 °C to generate a power density of about 100 mW/cm^2^ [4]. Recently, a much higher power density (about 600 mW/cm^2^ at 750 °C) has been demonstrated by using a multi-functional anode and iso-octane as fuel [5]. The main issue is the formation of carbon deposits on the anode, which is thermodynamically favorable under the reaction conditions [6]. At these temperatures organic fuels exist as vapors and therefore direct organic fuel SOFCs will not be discussed in this paper.

The most widely used fuel cells are based on proton exchange membranes (PEM), through which protons are transported (Figure 1a). The chemistry of anode and cathode reactions in the PEM hydrogen–oxygen regenerative fuel cell (RFC) is described by Equation 2 and Equation 3, respectively. Commonly used PEMs are generally based on sulfonated fluoropolymers such as Nafion^®^ 117 [7] that are stable and conductive up to 85–90 °C. Composite membranes based on both fluorinated and non-fluorinated materials, e.g., polysulfone polymers and inorganic proton conductors are used to achieve higher operating temperatures and a lower humidity [8]. Solid inorganic proton conductors (e.g., sintered zirconium phosphate) allow for increasing the working temperature up to 150–250 °C [9]. Only platinum group metal (PGM) electrocatalysts are stable enough in the low-pH environment of PEMs. Platinum is the best electrocatalyst for both hydrogen oxidation reaction (HOR) and oxygen reduction reaction (ORR), but it is very expensive. To reduce the Pt loading and therefore the cost for the electrocatalyst, Pt-containing alloys and structured nanoparticles, e.g., “core–shell” materials with less expensive metals are being investigated.

[2]
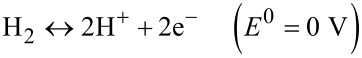


[3]
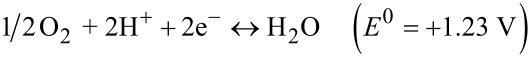


**Figure 2:**
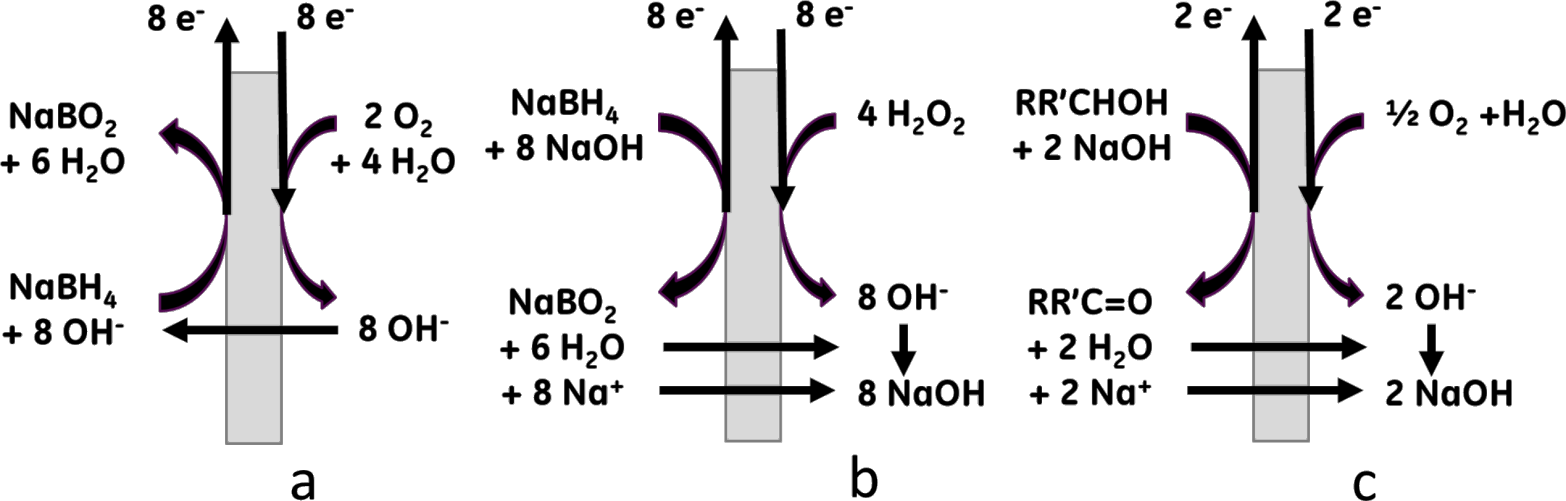
The electrochemistry of a borohydride liquid fuel cell with hydroxyl (a) and cation (b) exchange membranes and an alcohol fuel cell with cation exchange membrane (c).

Alkaline fuel cells are based on the transport of hydroxide ions through an anion-exchange membrane (AEM); the anode and cathode reactions are shown in Equation 4 and Equation 5, respectively (Figure 2b). They have the advantage of a lower redox potential for ORR in basic media (Equation 5). First such cells were developed at GE and used composite electrodes (a Pt black mixed with Teflon) and an AEM impregnated with 30% KOH [10]. An advantage of AEM fuel cells is that it is possible to use non-PGM electrocatalysts while classic PGM-oxide catalysts are less corrosion stable [11]. In general, AEMs have a lower conductivity and oxidative stability than PEMs [12]. The lower conductivity may be compensated for by the larger number of cationic sites, resulting in a high OH^−^ conductivity and power density. For example, an alkaline fuel cell utilizing a poly(vinylbenzyl(trimethylammonium hydroxide)) ion-exchange membrane showed a conductivity of 0.043 S/cm and current density of 0.72 A/cm^2^ at 0.6 V. Unfortunately, it was stable only up to 70 °C [13]. The sensitivity of AEMs towards nucleophilic attack in the working pH ranges and their reactivity with CO_2_ from air, which requires a scrubber or a closed system, limits their application.

[4]
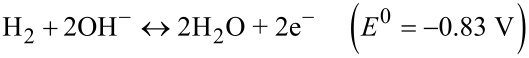


[5]
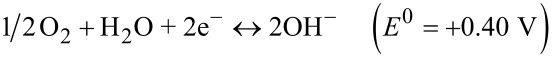


In general, in fuel cell systems oxygen is supplied by pumping air through the cathode, and hydrogen is stored on-site. Several types of hydrogen storage are currently considered: compressed gas, liquid hydrogen, metal hydrides (thermal release) or chemical hydrides (hydrolysis) [14].

The transportation and use of hydrogen as a fuel is limited by its physical properties (as the lightest element it has extremely low volumetric energy density) and safety issues (flammability and the formation of explosive mixtures with air). Hydrogen transportation is very expensive, therefore only few hydrogen fueling stations have been built so far, mostly in Europe (about 30) and the US (about 15). Though more fueling stations are planned, implementation of the hydrogen infrastructure will require an enormous capital investment. Compressed gas hydrogen (CGH_2_) storage in pressurized tanks (350 or 700 bar for mobile and 120 bar for stationary applications) is currently considered as the only practical option. It is the simplest method, which does not require expensive infrastructure and controls, but the system energy density is low, and there are safety hazards associated with high pressure and extreme flammability of hydrogen gas. Hydrogen can be stored in liquid form (LH_2_) in cryogenic tanks. This method has a higher energy density than CGH_2_. However, hydrogen liquefaction requires substantial energy (up to 30% of the lower heating value) and there are boil-off losses [15]. Hydrogen can be reversibly stored in metallic hydrides, e.g., intermetallic phases AB_5_ and AB_3_ [16], or complex hydrides, e.g., metal borohydrides M(BH_4_)*_n_* [17]. However, good hydrogen release kinetics and reversibility are inversely correlated with the storage capacity. Dehydrogenation of metal hydrides requires substantial thermal energy, which is technically challenging due to their low thermal conductivity.

The dehydrogenation of methylcyclohexane to toluene for both transportation and seasonal hydrogen storage was proposed 20 years ago [18]. Later the less volatile decalin/naphtalene couple with 7.3 wt % hydrogen content and a density of 64.8 kg-H_2_/m^3^ was also suggested [19]. In these systems, dehydrogenation can be done at ambient pressure, the heat transfer is not challenging, and the generated hydrogen is CO-free. A feasibility study of these systems showed the production cost of hydrogen to be $5.33/kg with the ratio energy generated/energy consumed of about 4 [20]. However, the hydrogen release from these compounds requires expensive PGM catalysts and high temperature (280 °C) resulting in large catalytic dehydrogenation reactor space and high cost requirements. The use of extended π-systems containing nitrogen atoms, such as *N*-alkylcarbazoles [21], enables a reduction in the heat and temperature of dehydrogenation (up to 200 °C), but the hydrogen content is lower. If all the heat is generated by the electrochemical oxidation of hydrogen, the overall system efficiency would be reduced from 55% to 44% which is still higher than the efficiency of internal combustion engines [22]. Alcohols constitute another class of liquid organic hydrogen carriers (LOHCs). Their hydrogen content is lower, but they can be dehydrogenated to aldehydes or ketones at much lower temperatures (80–90 °C) although side reactions, such as dehydration, are possible [23]. The rate of H_2_ generation from LOHCs is high enough to satisfy the demands of mobile applications, and the stability of the dehydrogenation catalyst exceeds several hundred hours [24]. The selectivity of dehydrogenation/hydrogenation reactions, a very important factor for energy storage, was reported to be over 99% for different classes of LOHCs and a promising cycling behavior was demonstrated [23–24]. The use of LOHCs, such as cycloalkanes, for hydrogen storage allows for the use of the existing liquid fuel infrastructure with relatively small modifications. However, in spite of the progress made in the last years, current technology is not yet close to meeting the revised targets of the United States Department of Energy (DOE) [25].

## Review

### Fuels for liquid fuel cells

Liquid-feed fuel cells can use different types of liquid fuels. Organic compounds that are liquids at ambient conditions can be used both neat and in the form of a solution. However, they are more often used in solution because of their flammability, toxicity and, most importantly, high crossover rates. Solid organic and inorganic compounds, e.g., NaBH_4_, can be used as a solution. Water is a natural solvent for organic and inorganic fuels, because it is produced at the cathode side, and it is the ion conducting medium in the majority of ion exchange membranes. Some of the proposed organic fuels are produced from renewable biomass, e.g., ethanol by fermentation of sugars, glycerol by transesterification of fats and oil triglycerides (biodiesel process), and furfural by hydrolysis of lignocellulose and agricultural byproducts (corncobs, wheat bran, etc.), which makes them especially attractive. In addition to individual compounds, mixtures can be used to improve electrode kinetics. Hydrazine, for example, was mixed with formic acid and methanol for that purpose [26].

In early works on liquid fuel cells several attempts to use hydrocarbons such as diesel and jet fuel were made. However, electrooxidation of hydrocarbons in low- and intermediate-temperature fuel cells turned out to be very difficult, and later the research focus was shifted to the oxidation of methanol in direct methanol fuel cells (DMFCs). Methanol has a higher energy density than liquid hydrogen and high theoretical fuel cell efficiency (Table 1). It was proposed as the basis for the “methanol economy” [27] as an alternative to the “hydrogen economy” based on hydrogen gas [1]. Alcohols with higher molecular weights, which contain C–C bonds, have an even higher energy density (Table 1) but their electrooxidation in fuel cells is not complete due to difficulty of activation of the C–C bonds and yields multiple intermediate products along with CO_2_ [28–30]. Polyoxomethylenedimethyl ethers (CH_3_O(CH_2_O)*_n_*CH_3_ (*n* = 1–8)) have been proposed as a fuel alternative to higher alcohols [31]. They have low vapor pressure and negligible toxicity, and undergo fast hydrolysis in the presence of acidic catalysts to release a mixture of methanol and formaldehyde that is oxidized several times faster than pure MeOH [31].

**Table 1:** Theoretical energy density and fuel cell efficiency for liquid fuels for fuel cells.

fuel	anode products	number of electrons	*E*^0^, V	energy density, Wh/L		η, %

				neat	solution	

liquid H_2_	H_2_O*	2	1.23	2350	—	83.0
H_2_ gas (70 MPa)	H_2_O*	2	1.23	1300	—	83.0
formic acid	CO_2_ + H_2_O	2	1.45	2103	1190 (10 M)	105.6
formate	CO_2_ + H_2_O	2	1.45	—	145 (1 M)	105.6
methanol	CO_2_ + H_2_O	8	1.17	5897	305 (2 M)	96.7
ethanol	CO_2_ + H_2_O	12	1.14	6307	915 (3 M)	97.0
ethanol	C_2_H_4_O	2	0.95	872	109 (3 M)	89.1
ethylene glycol	C_2_H_2_O_2_	4	0.87	1652	168 (1 M)	103.7
ethylene glycol	(COOH)_2_	8	1.09	4180	546 (2 M)	89.8
ethylene glycol	CO_2_ + H_2_O	10	0.87	5800	168 (1 M)	86.0
2-propanol	C_2_H_5_CHO	2	1.02	695	105 (2 M)	98.1
2-propanol	(CH_3_)_2_CO	2	1.07	750	114 (2 M)	98.2
glycerol	CO_2_ + H_2_O	14	1.21	5965	—	95.1
1,4-butanediol	C_4_H_6_O_2_	4	1.13	1361	—	89.9
2,4-pentanediol	C_5_H_8_O_2_	4	1.27	1105	—	111.8
furfural	CO_2_ + H_2_O	10	1.16	3915	—	76.3
cyclohexane	C_6_H_6_	6	1.06	1578	—	94.1
decalin	C_10_H_8_	10	1.09	1893	—	93.1
dodecahydro-*N*-ethylcarbazole	C_14_H_13_N	12	1.18	1715	—	n/a
ammonia	N_2_	3	1.17	—	1704 (35 wt %)	88.7
ammonia borane	NH_4_BO_2_	6	1.62	—	655 (2 M)	83.7
hydrazine hydrate	N_2_	4	1.56	4269	873 (4 M)	100.2
sodium borohydride	NaBO_2_	8	1.64	—	2940 (30 wt %)	93.4

In addition to alcohols, other organic compounds such as aldehydes (e.g., furfural) and acids (e.g., formic acid) may be used as liquid fuels for fuel cells. They have a high energy density (Table 1) and solubility in water. Aqueous solutions of sugars (glucose, sucrose, and lactose) were used in implantable bio micro fuel cells [32] but their energy density is too small to be used in large scale applications. Aqueous solutions of some inorganic compounds containing significant amount of hydrogen such as ammonia, hydrazine, alkali metal borohydrides MBH_4_ (M = Na, K) are also used as fuels. Theoretically, boron-nitrogen heterocycles proposed for hydrogen storage [33–34] can be used for this purpose.

In most cases the electrooxidation of fuels in fuel cells results in the formation of thermodynamically very stable and kinetically inert products. For instance, the electrooxidation of primary alcohols and formic acid generates CO_2_, and the oxidation of hydrazine releases N_2_. Such products cannot be directly converted back to starting fuels in a reverse reaction, and their regeneration requires an off-board multi-step process that is usually very energy demanding. For example, sodium borate can be regenerated to NaBH_4_ via ballmilling with MgH_2_ [35]. Another approach, which is a focus of the “Energy Frontier Research Center for Electrocatalysis, Transport Phenomena, and Materials for Innovative Energy Storage”, is to use partial electrooxidation of LOHC fuels to extract hydrogen (as protons and electrons) and form a stable dehydrogenated molecule, e.g., an aromatic or carbonyl compound (Equation 6) [36–37]. The overall reaction in the cell is described by Equation 7. The energy density of these systems is lower than those based on the full oxidation, but potentially they can be used for energy storage via electrochemical hydrogenation of the spent fuel (Equation 6 reverse). This approach is much simpler because it does not require an additional dehydrogenation catalyst nor a heat exchanger, and it has a higher energy density compared to hydrogen-on-demand designs that include the thermal decomposition of LOHCs in a catalytic reactor [38]. The spent (dehydrogenated) LOHC fuels can be re-hydrogenated either on-board (electrochemically) or off-board (electrochemically or chemically at a central plant). In the latter case, the fuel cells can be recharged by using the existing infrastructure for the delivery of liquid fuels.

[6]
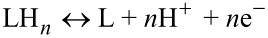


[7]
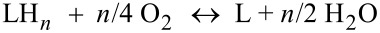


The theoretical open circuit potential (OCP) of electrochemical cells based on the reaction in Equation 7 is in the range of 1.06–1.11 V if the dehydrogenation product is an aromatic or carbonyl compound but only about 0.9 V if the product is an olefin [39]. For practical fuels, this results in theoretical energy densities of 1600–2200 Wh/L, which are comparable with that of liquid hydrogen (2540 Wh/L). In addition, the theoretical efficiency of organic fuel cells is higher than that of hydrogen (93–95% vs 83%) [39]. The partial electrochemical oxidation of fuels can also be used to produce valuable chemical products, e.g., acetaldehyde from ethanol or fine chemicals from glycerol, along with energy generation (“the chemical co-generation process”) [40–41].

#### Direct hydrocarbon fuel cells

Saturated hydrocarbons are attractive fuels for LFCs due to their extremely high energy density (9–10 kWh/L for full oxidation), abundance, low costs and the existing infrastructure. Early works on direct organic fuel cells were aimed at the use of liquid hydrocarbons (octane, decane, and, eventually, diesel fuel) as a fuel in phosphoric acid fuel cells. Linear hydrocarbons produced a higher current density on a Pt/PTFE anode in 95 wt % phosphoric acid at 175 °C while the addition of aromatic or branched hydrocarbons increased the anode overpotential [42]. On the other side, the presence of allyl hydrogen atoms in additives reduced the overpotential. A cell with a porous Pt/PTFE anode and cathode catalysts running on decane showed a maximum power density of 21 mW/cm^2^ (O_2_ cathode) and 17 mW/cm^2^ (air cathode) [43]. The addition of iso-alkanes to the fuel decreased the cell performance to about a third. Electrooxidation of hydrocarbon fuels in the presence of phosphoric acid requires a very high Pt loading (50 mg Pt/cm^2^), which makes this approach unfeasible. Lower hydrocarbons, e.g., propane [44–45] and cyclohexane [46–47] were used as fuels for PEM fuel cells but in the vapor form.

#### Direct alcohol fuel cells

Both monohydric and polyhydric alcohols have been proposed and used as fuel for LFCs in aqueous solution. The most extensively studied DMFC technology based on the reaction in Equation 8 has been reviewed in multiple papers [48–56], and will be discussed here only for comparison properties.

[8]
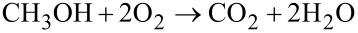


Thermodynamic analysis of fuel cells based on C_1_–C_5_ alcohols showed that these cells have OCVs that are only 10–100 mV lower than hydrogen fuel cells but exhibit a higher theoretical efficiency [57–58]. By combination of parameters such as efficiency, OCV and specific energy, only MeOH and EtOH can compete with hydrogen as a fuel at temperatures below 75 °C while in the intermediate temperature range (up to 300 °C) C_1_–C_3_ alcohols are preferred [58].

#### Direct ethanol fuel cells

Ethanol is a renewable, inexpensive feedstock and as a fuel has a very high energy density and theoretical cell efficiency if fully oxidized to CO_2_ and water (Table 1). Therefore, ethanol based LFCs are considered for mobile applications [59]. However, breaking the C–C bond is extremely difficult and the CO_2_ yield is usually low [57]. This has been explained by the higher energy barrier of the key step of CO formation due to the presence of surface O and OH species [60]. In acidic media two reactions (Equation 9 and Equation 10) are dominant. The formation of acetaldehyde occurs at lower potentials (<0.6 V vs RHE) while acetic acid is produced under consumption of a water molecule at higher potentials (>0.8 V vs RHE) [61]. The product of another two-electron oxidation reaction, ethane-1,1-diol, is formed in substantial quantities when Pt/C is used as a catalyst and is also present as a minor product in the presence of bimetallic catalysts [62]. The selective oxidation of EtOH to ethylacetate, without the formation of CO_2_, takes place in sulfuric acid solution at a reduced SO_2_-treated porous Pt black anode [63].

[9]
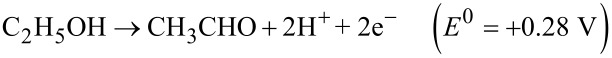


[10]
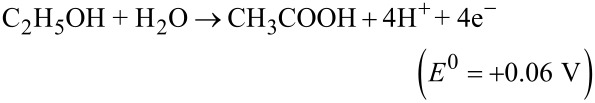


The kinetics of ethanol electrooxidation on Pt-based anodes in acidic media is much slower than that of hydrogen and results in high fuel cell overpotentials (usually 0.3–0.6V) [64]. This is partially compensated by lower ethanol crossover through acidic membranes and lesser cathode poisoning [65]. PtRu and PtSn catalysts were much more active than pure Pt [57]. Double-layered anode catalysts consisting of 45 wt % Pt_3_Sn/C and PtRu black electrocatalysts showed an improvement of about 40% in power density (up to 96 mW/cm^2^) and a higher yield of acetic acid [66]. The addition of Ni to binary Pd–Sn alloys increases the electrocatalytic activity [67]. Intermetallic phases of Pt with In, Sn, Pb, Bi, and As were studied as electrocatalysts for the oxidation of ethanol [68]. In contrast to its inactivity towards MeOH oxidation, the PtBi phase was electrocatalytically active in EtOH oxidation while the PtBi_2_ phase and other PtM*_x_* phases with a ratio Pt:M (M = Sn) different from 1 were inactive [68].

Low overpotentials for the oxidation of EtOH were achieved with Pt*_n_*(SnO_2_)/C (*n* = 1, 3, 9) electrocatalysts. A fuel cell using 2 M EtOH, a Nafion® 117 membrane and a Pt/C cathode catalyst reached a peak power density of 127 mW/cm^2^ for *n* = 3 at 100 °C [69]. Acetaldehyde and acetic acid were the major products, and the yield of CO_2_ was below 7%. Acetic acid is not electroactive under the fuel cell conditions while acetaldehyde can be used as a fuel although it generates half the power [69]. Addition of acetaldehyde to ethanol impairs the performance of the fuel cell.

Apart from the development of more active catalysts that are less sensitive to CO poisoning, another approach is to increase the operation temperature. To this end, a composite silica/Nafion® membrane was used at 145 °C to reach a maximum power density 110 mW/cm^2^ with 1 M EtOH feed [70]. Under these conditions CO_2_ becomes the major product along with a smaller amount of acetaldehyde. Contrary, a vapor-fed fuel cell with H_3_PO_4_-doped polybenzimidazole (PBI) membrane and similar catalysts produced mostly acetaldehyde at a higher temperature (170 °C) [71–72].

Higher primary alcohols starting from 1-propanol exhibit even slower kinetics in acidic media and, therefore, are not considered as promising fuels [71,73].

The operation of alkaline ethanol LFC has potential benefits compared with PEM LFC including faster kinetics of both ethanol oxidation and oxygen reduction in basic media and lower fuel crossover due to a reversed electro-osmotic effect of anion movement in the membrane. The major product of EtOH electrooxidation in alkaline solution is acetate (Equation 11)

[11]
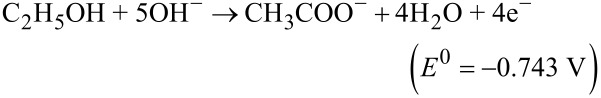


The product distribution of electrochemical ethanol oxidation in basic media depends on the catalyst. A Pt catalyst generates acetate that converts to ethylacetate at higher EtOH concentrations along with some CO_2_. Acetate is formed at a Pd catalyst with very high faradaic efficiency while ethylacetate is the only product at a Ag catalyst [74]. The addition of a base (NaOH or KOH) in the concentration of at least 1 M to ethanol solutions is necessary to provide good conductivity. It was found that for 2 M fuel and 3 M KOH the current density was similar for methanol or ethanol but the ethanol cell exhibited a slightly higher voltage [75]. A cell with a non-platinum HYPERMEC^TM^ (Acta) anode and cathode catalyst and Tokuyama® AEM using 3 M EtOH and 5 M KOH showed an OCV of about 900 mV and a peak power density of 60 mW/cm^2^ [76]. Replacing the cathode catalyst with Pd_3_Au/CNT increased the power density to 185 mW/cm^2^ [77].

A thermally stable PBI membrane doped with 2 M KOH was used as AEM in a direct ethanol LFC to expand the operational temperature range [78]. The cell, equipped with a 45% PtRu anode catalyst and a 40% MnO_2_/C cathode catalyst, achieved a maximum power density of 30 mW/cm^2^ using a 2 M EtOH/2 M KOH fuel mixture, but the cell performance quickly degraded (more than 50% after 200 h) [78].

A cell with hydrogen peroxide as the oxidant and a non-platinum anode showed 44% increase in power density (160 mW/cm^2^ at 80 °C) compared to a similar cell with an air cathode [79]. In an innovative cell design proposed by T. Zhao at el. [80], anode (ethanol in a basic media) and cathode (H_2_O_2_ in an acidic media) are separated by a cation exchange membrane. The cell, equipped with 15 µm Nafion® N211 PEM and PtNi/C electrocatalysts, reached a peak power density of 360 mW/cm^2^ at 60 °C, which is a substantial increase compared to the state-of-the-art direct ethanol fuel cells. A high theoretical OCP (2.52 V) is rendered by both the oxidation of ethanol to acetate and the neutralization reaction that gives sodium sulfate as a by-product (Equation 12). Though the highest cell voltage was measured for a fuel concentraion of 5 M , the cell optimal performance was reached for 3 M EtOH and 5 M NaOH [80].

[12]
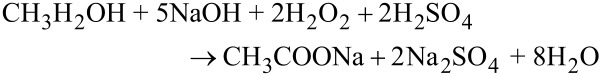


#### Direct isopropanol fuel cells

Isopropanol (IPA) is relatively inexpensive, has a low toxicity and is miscible with water. Electrooxidation of IPA on different catalysts in both acidic [81–82] (Equation 13) and alkaline [83–87] (Equation 14) media has been studied. In both reactions acetone is the single oxidation product but at high potentials the formation of CO_2_ was detected [88]. At low current densities, the formation of H_2_ as a result of IPA dehydrogenation on a Pt catalyst was reported [89]. In acidic solutions only Pt and PtRu are used as electrocatalysts [81–82], while in alkaline solutions the catalyst selection is wider. At high pH values Pt is not the most active electrocatalyst, and Pd is at least at par or even superior [83–84]. Although Au is less active than Pd and Pt in a pure form [83], its addition to Pd in the ratio of 1 to 4 increases the catalyst activity and stability for IPA oxidation [85]. Ni metal supported on carbon catalyzed the IPA electrooxidation [28], which was attributed to the formation of surface β-NiOOH species [87].

[13]
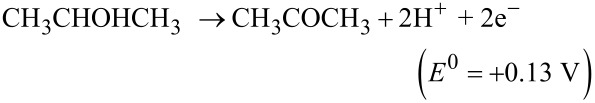


[14]
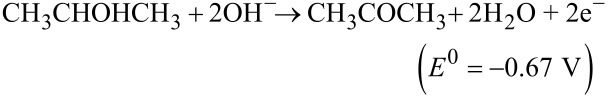


A fuel cell with a Ni/C anode catalyst and a Nafion® 117 PEM showed a higher OCV for 2 M IPA in water than for MeOH but the current density was low (about 1 mA/cm^2^) and the cell voltage dropped with time [28]. It is noteworthy, that at 80 °C the Ni/C catalyst was more active than the Pt/C catalyst, but exhibited a worse fuel cell performance, presumably due to the catalyst poisoning [28]. The use of PtRu anode catalysts, a Pt cathode catalyst and a Nafion® 112 PEM resulted in a higher peak power density (80 mW/cm^2^) and relatively low crossover current (approximately 30% of that for MeOH) [90]. It was found that the cell performance was best with 1 M IPA at 60 °C [90]. A similar cell equipped with a sulfonated poly(ether ether ketone) (SPEEK) membrane using neat IPA as a fuel delivered 97 mW/cm^2^ at 60 °C but a stable performance was observed only for low current densities (10 mA/cm^2^) [91]. The crossover of neat IPA through the SPEEK PEM was about the same as for the 1 M solution due to lower swelling [91]. A similar LFC operating on 2 M IPA at 90 °C exhibited a high OCV (0.86 V) and achieved a peak power density of 128 mW/cm^2^ [89]. The cell voltage was ca. 200 mV higher, and the electrical efficiency (59%) was 27% higher than that of the cell operating on methanol. However, the cell performance sharply dropped when the current density exceeded 200 mA/cm^2^, which is attributed to catalyst poisoning by acetone or products of deep oxidation of IPA [89].

The use of neat IPA with liquid 5 M KOH electrolyte and commercial Pt/C catalyst provided a peak power of 22 mW/cm^2^ [92], which was higher than for a cell with a PtRu catalyst and a PVA/TiO_2_ membrane using 2 M IPA/2 M KOH fuel (16 mW/cm^2^) [93]. A mixture of methanol and 2-propanol that has a low electrooxidation onset and higher oxidation current densities than single alcohols was proposed as a fuel [94].

1-Methoxy-2-propanol was used as a fuel in a PEM fuel cell showing a high OCV (0.71 V) but the cell performance degraded faster than with IPA [95].

#### Direct ethylene glycol fuel cells

The theoretical energy density of ethylene glycol (EG) is comparable to those of methanol and glycerol (Table 1), However, the complete electrooxidation of EG to CO_2_ and H_2_O, a ten-electron process, has not been achieved [96]. The electrochemical oxidation of EG on Pt yields a mixture of products: glycolic acid and CO_2_ in acidic media, and glycolate, oxalate and carbonate in alkaline media [97]. Glycol aldehyde and oxalic acid were also detected in HClO_4_ solution [98]. The catalyst is poisoned by intermediates that have been identified as CO-like species [99]. The electrochemical oxidation of EG on Pt–Sn catalysts is a four-electron process, which corresponds to the formation of glycolic acid, a major product determined by chromatography (GCMS) [100].

An acidic EG fuel cell using a 100 µm nanoporous proton-conducting membrane and a Pt–Ru anode catalyst demonstrated a peak power density of 300 mW/cm^2^ for the anolyte containing 2 M EG and 3 M H_2_SO_4_, which was higher than a cell with a Nafion® 115 membrane [101]. Replacing the sulfuric acid with triflic acid decreased the anodic overpotential and increased the maximum power density to 320 mW/cm^2^ at a lower acid concentration (1.7 M) [102]. In a 10-cell stack with the same membrane fed with 0.5 M EG in 1.7 M triflic acid solution, the power density was 120 mW/cm^2^ and two major by-products (glycolic and oxalic acids) were identified [103]. Discharging without EG feeding consumed the by-products almost completely; this shows the possibility of a complete EG oxidation to CO_2_ [103].

A basic EG fuel cell with a 28 µm Tokuyama AEM was tested with a PdNi/C anode catalyst and a non-Pt cathode catalyst at different concentrations of EG (0.5–3 M) [104]. It was found that 1 M EG was the optimal EG concentration. The maximum power density reached with 7 M KOH was 67 mW/cm^2^ at 60 °C [104]. The use of an alkali-doped polybenzimidazole membrane resulted in the increase of the maximum power density for the same fuel composition to 80 mW/cm^2^ (at 60 °C) and 112 mW/cm^2^ (at 90 °C), which was 2–3 times higher than for the same cells fueled with MeOH and EtOH [105]. An interesting concept of an EG fuel cell using a LaSr_3_Fe_3_O_10_ ceramic disk as a membrane and ORR catalyst was demonstrated in a cell with 10 wt % EG, 10% KOH and FeCoNi/C anode catalyst to give oxalic acid as a major product (Equation 15) and a power density of 27 mW/cm^2^ [106].

[15]
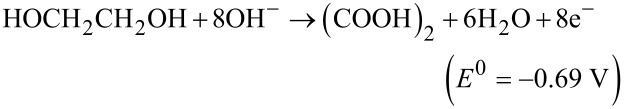


#### Direct glycerol fuel cells

Glycerol as a nontoxic fuel for fuel cells was proposed in 1964 [107]. Glycerol is the major product in biodiesel production by transesterification of plant oils and animal fats. Although it is used as a raw material in the chemical industry and animal feed, its market is saturated thus limiting the expansion of biodiesel [108]. Having a high energy density (Table 1), glycerol is a promising fuel. However, as in the case of other C_2_ and higher alcohols, the total oxidation has not been demonstrated.

The electrooxidation of glycerol in acidic media on a Pt/C electrode gives a mixture of products with glyceraldehyde as the major one. The addition of bismuth as a saturated solution redirects the reaction towards 100% selective formation of dihydroxyacetone [109]. The bulk electrolysis of glycerol in 0.1 M NaOH on Ni/C and NiCo/C anodes gives formate, glycolate and glycerate as major products [110]. The electrooxidation of glycerol on the Au/C anode in alkaline LFC yields predominantly salts of tartronic, glyceric, mesoxalic and oxalic acids with a faradaic efficiency of 53–58% [111]. The electrooxidation of glycerol on an optimized Ru–Ni catalyst was 3–4 times faster than the oxidation of ethanol [112].

A direct glycerol fuel cell fed with glycerol (1 M) in KOH (4 M) using a polybenzimidazole (PBI) membrane impregnated with KOH and PtRu/C and Pt_3_Sn/C anode catalysts showed a peak power density of 18 mW/cm^2^ at 60 °C, which decreased as the temperature increased to 90 °C [113]. Pd-based electrocatalysts showed a much higher activity than Pt-based ones, e.g., PtRu, which is widely used in DMFCs [114]. An active alkaline fuel cell running on 5% glycerol and using Pd catalyst supported on multi-wall carbon nanotubes generated 80 mW/cm^2^ peak power at 80 °C. The product mixture included formate and carbonate [114].

The use of crude glycerol from the biodiesel process in an AEM fuel cell has been reported [115]. The use of dealloyed PtCo nanoparticles on a carbon nanotube support surface in such a cell allowed for a peak power density of 268.5 mW/cm^2^ at 80 °C with the anode catalyst loading of 0.5 mg Pt/cm^2^ [116].

### Fuel cells with other oxygenated fuels

Abundant and energy dense sugars are natural fuels for bio (microbial or enzymatic) fuel cells using whole cells or isolated redox enzymes to catalyze the oxidation [117]. These cells demonstrate very low power densities and will not be discussed in this paper. PGM catalysts exhibit low catalytic activity in electrooxidadion of carbohydrates. The oxidation of glucose in 1 M KOH in alkaline liquid fuel cells with a PtRu electrocatalyst generates gluconic acid (two-electron process) and 1. 4 mW/cm^2^ peak power [118]. The use of a Pt/C anode with a cobalt phthalocyanine cathode in an alkaline cell with a Tokuyama membrane provided a maximum power density of 2.3 mW/cm^2^ in 0.5 M glucose/0.5 M KOH solution [119]. Increasing the KOH concentration to 7 M, in combination with a PdNi anode and a non-platinum HYPERMEC^TM^ cathode (Acta), resulted in a substantial increase in power density to 38 mW/cm^2^ at 60 °C [120]. Sorbitol and xylose were also used as fuels but demonstrated slightly slower kinetics [121–122]. The performance of the fuel cells with carbohydrate fuels significantly decreased with time, which was partially attributed to sorbitol and glucose crossover-poisoning the Pt/C cathode [121].

L-Ascorbic acid (AA, also known as vitamin C) has been proposed as a fuel for liquid-fed fuel cells because it is benign, renewable, inexpensive, and highly soluble in water (330 g/L) [123]. PGM catalysts are not necessary for the anodic oxidation of AA, e.g., a polyaniline-based anode produced 4.3 mW/cm^2^ at 70 °C with liquid fuel of 1 M AA in 0.5 M H_2_SO_4_ [124]. The use of treated carbon black (Vulcan X72) produced a four-fold increase of the peak power density [125]. Dehydroascorbic acid was the only electrooxidation product detected [125], which sets the theoretical energy density of 110 Wh/L at the maximum concentration in water. The acidic nature of AA as a fuel reduces its crossover through acidic membranes. Unfortunately, the low power of such fuel cells makes them useful only to long-term portable or implantable applications.

#### Direct formic acid fuel cells

Formic acid has a high OCP (Table 1), and the fact that it is liquid at room temperature and non-toxic in diluted solutions makes it an attractive fuel candidate [126]. The crossover flux of formic acid through PFSA membranes is less than that of MeOH [127], which allows for the use of much higher concentrations (10×). It results in higher energy densities compared to DMFC in spite of the higher theoretical energy density of methanol (Table 1). Electrooxidation of formic acid is described by Equation 16. A parallel undesirable reaction pathway leads to the formation of adsorbed CO species, which are then oxidized to the final product, CO_2_ [128]. All known anode electrocatalysts contain Pt or Pd, though the pure metals cannot be used due to surface poisoning with CO. It was shown that addition of Ru (up to 50 mol %) decreases the quasi-steady-state level of adsorbed CO [128]. Replacement of ruthenium with gold in the bimetallic catalyst increases catalytic activity that results in higher cell voltage [129]. A series of intermetallic phases of Pt with In, Sn, Pb, Bi, and As was identified as promising electrocatalysts for oxidation of formic acid with PtBi_2_ being the most active [68]. The use of a Pt_4_Mo alloy increases the reaction rate by more than one order of magnitude compared to pure Pt supposedly due to the formation of hydrous molybdenum oxide that reduces the surface poisoning by adsorbed CO [130].

[16]
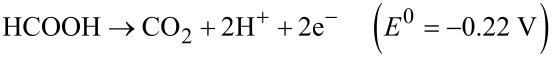


Palladium-based electrocatalysts deliver higher power densities compared to platinum-based ones. Fuel cells with a Pd black anode catalyst and 3 M HCOOH reached a peak power density of 375 mW/cm^2^ at 50 °C [131]. The power density is independent of the formic acid concentration up to 10 M, which allows for high energy densities (Table 1). Unfortunately, limited life-testing data indicates that the catalyst deactivates within several hours, and the rate of deactivation increases with the acid concentration. However, the loss of activity is reversible, and it can be restored by pulsing the potential [131]. In contrast to Pt, the addition of a second component (Ru, Au) decreases the catalyst performance of Pd black [132]. The use of a Pd/C catalyst results in lower power densities (145–170 mW/cm^2^ depending on the loading), but a more stable performance [133]. The alloying of Pd with Sb [134] and Bi [135] in carbon-supported catalysts increased the power density, which reached 260 mW/cm^2^ for Pd–Bi/C catalyst with 5 M HCOOH. A similar effect was achieved by deposition of bismuth on the Pt nanoparticles by irreversible adatom adsorption [136]. It was claimed that the addition of Ni to Pd/C improves the catalyst performance and stability [137]. Different type of supports have been tried to replace traditional carbon support, e.g., Vulcan XC-72. The addition of more corrosion-resistant ZrC to XC-72 carbon (1:1) provided a narrower particle size distribution and a better dispersion on the surface and resulted in a higher activity during formic acid oxidation [138]. Nanocomposite-based on Pd/MnO_2_/nanolamella-graphene sheets showed an activity that was about six times higher than that of a traditional Pd/C catalyst [139]. Although the peak power density for supported Pd-based catalysts is lower than for Pd black, the palladium utilization and specific power density (mW per mass unit) are much higher.

The flux of formic acid across Nafion® membranes increases with concentration. It is only about half of that of MeOH, but the resulting crossover current is much lower (by about a factor of 6) due to the smaller number of participating electrons (8 vs 2, compare Equation 8 and Equation 16) [140]. Due to their high power density, low crossover and an the insufficient stability of the electrocatalysts, the development of direct formic acid fuel cells currently targets small scale portable applications including microcells [126].

The electrooxidation of the formate anion in alkaline media (Equation 17) combined with ORR reaction (Equation 5) is used in a formate alkaline fuel cell [141]. A fuel cell equipped with an AEM demonstrated a high OCV (0.93 V) and a high peak power density (125 mW/cm^2^ for a 1:1 mixture of KCOOH and KOH at 60 °C). It was shown that the formate oxidation reaction does not depend on the pH value in a range between pH 9 and 14, so formate fuel can be used without added hydroxide [142]. However, the power density increases with the KOH concentration and drops substantially without the base. A concentration of 1 M KOH seems to be optimal [141]. This limits the energy density of this system (Table 1). The increase of the working temperature to 120 °C and of the KCOOH concentration to 6 M in a similar cell with a Ag cathode catalyst resulted in higher power density (160 mW/cm^2^) [143]. Alkaline media is favorable for faster electrooxidation kinetics of the formate anion, and formate salts are non-hazardous and easy to transport. However, the reaction (Equation 17) generates alkaline metal carbonates as a waste, which decreases the attractiveness of this approach.

[17]
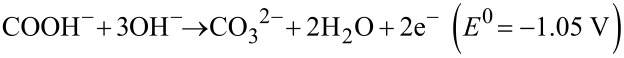


The mixture of formic acid and formate was proposed as a fuel for a direct fuel cell [144]. In the presence of formate the oxidation potential of formic acid was shifted in the negative direction and the oxidation current increased. In this case only formic acid was oxidized.

### Inorganic fuel cells

#### Direct ammonia fuel cells

The nitrogen hydrides, ammonia and hydrazine, are attractive fuels for direct fuel cells because potentially they can be cleanly oxidized to water and nitrogen [145–146]. Ammonia cannot be used directly in acidic PEM fuel cells due to a sharp drop in membrane conductivity (ammonium salt formation) and catalysts poisoning [147]. In an early work, a fuel cell using aqueous potassium hydroxide and PTFE-bonded Pt black supported on graphite electrodes, combined with an air cathode, demonstrated power densities of 50 mW/cm^2^ at 0.5 V at 120 °C [148]. A fuel cell with a Cr-decorated Ni anode, a MnO_2_/C cathode, and an Amberlite–based membrane using 35% ammonia solution showed a peak power density of about 9 mW/cm^2^ at room temperature, which was nevertheless higher than that for hydrogen fuel under the same conditions [149]. The main challenge of direct ammonia fuel cells is the development of robust anode electrocatalysts. It was found that PtRu/C is much more active than individual metals but still achieves only current densities below 30 mA/cm^2^ [150]. Accumulation of adsorbed nitrogen species on the catalyst surface causes catalyst degradation. Another drawback of these cells is the ammonia flux through the anion exchange membrane [150].

#### Direct hydrazine fuel cells

The concept of a direct hydrazine fuel cell was developed in the 1960s [151–152]. Hydrazine can be electrochemically oxidized as the hydrazonium cation N_2_H_5_^+^ in acidic and neutral (due to hydrolysis) media (Equation 18) or as a neutral molecule in basic solutions (Equation 19). The overall cell reaction generates only nitrogen and water, with a standard OCP of 1.56 V.

[18]
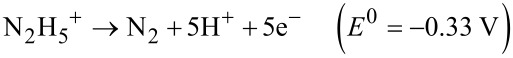


[19]
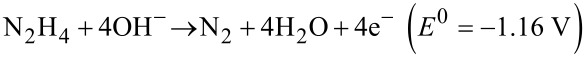


Theoretically hydrazine fuel has a very high energy density (Table 1) but the need to use diluted solutions limits the energy density, e.g., to 340 Wh/L for 10 wt % hydrazine hydrate solutions used in a PEM fuel cell [153]. A cell with 60 wt % Pt on carbon catalyst and Nafion® 117 membrane showed a high OCV (about twice as high as with MeOH) but the higher internal resistance limited the power density to about 100 mW/cm^2^ [153]. It was found that the catalytic decomposition of hydrazine on Pt generates both hydrogen and ammonia (via two different pathways), which reduces the OCV. In addition, a substantial flux of hydrazine and ammonia through the PEM, causing degradation of the cathode, was observed [153]. These issues shifted the research focus exclusively to alkaline hydrazine fuel cells in the subsequent years [154].

The search for hydrazine oxidation electrocatalysts is complicated because of the competing reactions leading to a decomposition of hydrazine, which are catalyzed by the same catalysts [155]. Platinum in alkaline media is less active than Ag, Ni and Co, and this opens a pathway to PGM-free fuel cells [156]. The activity of Ag and Pd nanoparticles on carbon was comparable [157]. Nickel-0based electrocatalysts are the most active for hydrazine oxidation. An alloy with the composition Ni_0.6_Co_0.4_ was about 6 times more active than the pure Ni catalyst [155]. The design space of binary Ni–M (with M = Mn, Fe, Zn, La) and ternary Ni–Mn–Fe and Ni–Zn–La compositions was explored by using the combinatorial approach. The compositions Ni_0.87_Zn_0.13_ and Ni_0.9_La_0.1_ prepared by spray pyrolysis were the most active showing power densities of 486 and 459 mW/cm^2^, respectively [158]. The enhanced electrocatalytic performance of the latter may be explained by the formation of a LaNi_5_ coating on the surface [159]. More than 2000 h of continuous operation at 70% efficiency were demonstrated with a cell with a nanotextured Cu–Ni anode, although with a low current density (14 mA/cm^2^) [160].

The use of hydrogen peroxide as an oxidant (Equation 20) in a direct hydrazine fuel cell fuel cell delivers a high OCP (2.13 V), which can be even higher when the anode is basic and the cathode is acidic. Thus, a cell, with Ni–Pt/C anode and Au/C cathode catalysts, 10 wt % hydrazine/15 wt % NaOH anolyte and 20 wt % hydrogen peroxide/5 wt % H_2_SO_4_ catholyte, had a high OCV (1.75) V and showed a very high peak power density (1.02 W/cm^2^ at 80 °C) [161]. A higher temperature improves the performance of the cathode but has little effect on the anode [161]. An electrocatalyst consisting of dealloyed nanoporous gold leaves demonstrated activities toward both hydrazine oxidation and hydrogen peroxide reduction that were about 22 times higher than a commercial Pt/C electrocatalyst at the same loading [162].

[20]
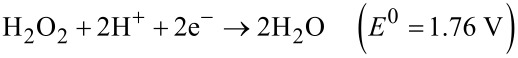


In an attempt to increase the conductivity and stability of the AEM, a composite membrane of a hydroxyl conducting quaternary ammonium polymer confined in a pre-treated PTFE matrix was prepared through in situ polymerization. It had a conductivity of 0.049 S/cm at room temperature, which resulted in a peak power density of 110 mW/cm^2^ [163].

The main drawback of direct hydrazine fuel cells is the high toxicity of N_2_H_4_ and its derivatives [164]. Less toxic hydrazine derivatives such as carbohydrazide (N_2_H_3_)_2_CO have been proposed to solve this issue [165]. Carbohydrazide has 71% of the capacity of hydrazine, it is miscible with water, and it can be electrochemically oxidized in the presence of inexpensive cobalt porphyrines [165].

#### Direct borohydride fuel cells

The high OCP and energy density of direct fuel cells with anodes that contain borohydride (tetrahydroborate, BH_4_^−^) salts make them attractive for portable applications and stimulated recent research in this area [166–167]. Only borohydrides of alkali metals (except Li) are stable towards hydrolysis at high pH values. Electrooxidation of the borohydride anion in alkaline media is an eight-electron reaction (Equation 21). When coupled with ORR (Equation 5, net reaction in Equation 22), the theoretical cell OCP is very high, about 400 mV higher than the OCP of a fuel cell with a hydrogen anode (Table 1). However, the observed OCV of the direct borohydride fuel cell is much lower, presumably because it is a mixed potential of the reaction in Equation 22 and the thermodynamically favorable reaction in Equation 4, in which hydrogen is generated by the competing hydrolysis of borohydride (Equation 23) [166]. If hydrogen is oxidized fast, e.g., by using active porous electrodes, the total number of transferred electrons is still eight as was shown by rotating disk electrode experiments on an Au electrode [168].

[21]
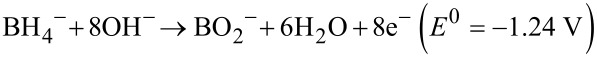


[22]
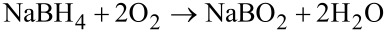


[23]
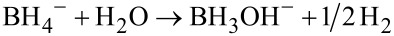


The electrooxidation of borohydride anions is a multi-step electron transfer process with competing parallel chemical reactions, and its mechanism is not fully understood [169]. The number of electrons removed from the BH_4_^−^ ion depends on the anode electrocatalyst, the concentration of sodium borohydride and the ratio [OH^−^]:[BH_4_^−^]. For a ratio of about 4.4, the reaction is described by Equation 21, while for lower ratios the reaction in Equation 23 takes place predominantly, which leads to a decrease in the number of electrons [170]. It was shown that on a Pt/C catalyst the borohydride anion is oxidized by an eight-electron reaction at concentrations below 1.5 M, and by a six-electron reaction under H_2_ evolution at concentrations above 2 M at a more negative electrode potential (−1.38 V) [171]. The electrode potential increases even further (*E*^0^ = −1.65 V) for a four-electron reaction but this increase does not compensate for the loss of capacity [172] Non-PGM cathode catalysts for direct borohydride fuel cells based on Ni, Co and Mn oxides show activities comparable or sometimes higher than conventional Pt/C, with LaNiO_3_ being the most active [173].

Charge neutrality during electrooxidation of BH_4_^−^ ions (Equation 21) can be achieved in two ways: (1) by the movement of cations (Na^+^) across a cation exchange membrane (CEM) (Figure 2b,c), or (2) by the movement of anions (OH^−^) across an anion exchange membrane (AEM) (Figure 2a). Therefore two basic designs using CEM and AEM are known in the literature [166–167]. AEM-based designs offer simpler processes where borohydride, borate and alkali metal ions are confined in the anode compartment, stabilizing the pH of the anolyte. However, AEMs are not stable in concentrated alkali especially at elevated temperatures [12–13]. Another issue of direct borohydride fuel cells with AEMs is crossover of BH_4_^−^ ions to the cathode, which substantially reduces the cell efficiency and poisons the cathode catalyst.

A fuel cell with a Laves phase AB_2_ Zr–Ni based alloy and Pd/C as the anode catalyst, a Nafion® NRE-211 membrane and Pt/C as the cathode catalyst delivered a power density of 290 mW/cm^2^ at 60 °C [172]. The NaBH_4_ utilization was only 51% but increased with lower temperatures. Combining a borohydride electrolyte with a mixed anode (Zn + LaNi_4.7_Al_0.3_) and a MnO_2_ cathode catalyst allowed for an increased cell capacity (up to 1800 mA/g for the anode) and an increased peak power compared to a Zn/air cell [174].

Replacing the ion exchange membranes with a fiber separator made of inexpensive polymer materials (polypropylene or polyamide) and allowing the free movement of all ions resulted in substantial increase in the power density compared to Nafion® CEMs. A cell with non-PGM catalysts (LaNiO_3_ in the cathode and Co(II) oxide in the anode) delivered a peak power of 663 mW/cm^2^ at 65 °C [173].

Another promising modification of the direct borohydride fuel cell is an all-liquid cell with hydrogen peroxide cathode. The standard electrode potential of the H_2_O_2_ cathode is 530 mV higher than that of the oxygen cathode, with a similar dependence on the pH value. A cell based on the reactions in Equation 21 and Equation 24 has a high OCP (2.14 V) and a high theoretical energy density (2580 Wh/kg), as well as a simpler heat management compared to fuel cells with gas electrodes. The charge is balanced by the transport of Na^+^ ions across a CEM.

[24]
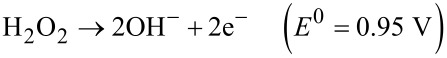


The all-liquid direct borohydride cell with a mischmetal MmNi_3.55_Al_0.3_Mn_0.4_Co_0.75_ anode and a 60 wt % Pt/C cathode catalyst showed a peak power density of 350 mW/cm^2^ at 70 °C when fed with 10 wt % NaBH_4_ in 20 wt % NaOH to the anode and 15 wt % H_2_O_2_ (pH 0) to the cathode [175]. The cell voltage at maximum power was 1.2 V and decreased with increasing catholyte pH value. Therefore, to obtain a steady cell performance it is necessary to maintain a high pH in the anolyte and a low pH in the catholyte, thus consuming also a base and an acid (Equation 25) [175]. The cell performance was optimized with 8 wt % NaBH_4_ in the anolyte and with 2 M H_2_O_2_ in 1.5 M H_2_SO_4_ in the catholyte [176]. A similar composition of fuel (10 wt % NaBH_4_ in 20 wt % NaOH, 15 wt % H_2_O_2_ in 1 M sulfuric acid) produced 410 mW/cm^2^ at 80 °C [177].

[25]
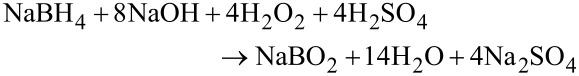


The sputtering of metals on a carbon cloth provides well-dispersed nanoscale particles with high catalytic activity resulting in a peak power density of 680 mW/cm^2^ at 60 °C, with Pd and Au as anode and cathode catalysts, respectively [178].

#### Direct ammonia borane fuel cells

Ammonia borane NH_3_BH_3_ (AB) has 19.6% hydrogen, is easily soluble in water and reasonably stable towards the hydrolysis in the absence of catalysts, which makes it a promising fuel for liquid-fed fuel cells [179]. AB is electrochemically oxidized in alkaline media to environmentally benign products (Equation 26). An AB fuel cell has OCV 1.616 V and theoretical specific energy 2113 Wh/kg at the maximum concentration. In practice, solutions containing a base have a lower specific energy, of about 30% of the theoretical value (Table 1). In principle, ammonia, one of the products of the reaction in Equation 26, can be oxidized in alkaline media [146] thus increasing the cell capacity. However, the large potential difference (450 mV) and slow kinetics make this difficult.

[26]
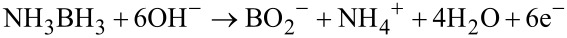


Similarly to sodium borohydride, the hydrolysis of AB decreases the cell voltage and coulombic efficiency [179]. A fuel cell using 46.6 wt % Pt on Vulcan XC-72 reached a maximum power density of 185 mW/cm^2^ [180]. Gold electrocatalysts having low activity towards hydrolysis turned out to be more efficient catalysts than platinum. Thiourea, a known hydrogen evolution inhibitor, was also used as an additive to increase the coulombic efficiency [181]. Nanoporous gold electrodes prepared by extracting Ag from an AgAu alloy catalyze the oxidation of AB at a potential more negative by 280 mV, and current densities 5 times higher than those obtained with a pure Au disk electrode [182]. It was found that smooth Cu metal is as good an electrocatalyst as Pt nanoparticles for the oxidation of AB [183]. Nanostructured Cu with petal-like structures possessed a much higher electrocatalytic activity and when used as an anode in a fuel cell with a commercial air cathode provided a power density of about 1 W/cm^2^ with an OCV of 1.26 V at room temperature [183].

The recent development of inexpensive Cu electrocatalysts is a substantial progress towards the use of AB as a fuel in practical fuel cells. However, there are a number of issues to be solved including low efficiency due to hydrolysis, fuel and products crossover, product crystallization in MEA and the high cost of AB. Although a one-pot method for the conversion of the thermal decomposition products of AB back to AB by a treatment with hydrazine was recently developed [184], the regeneration of AB from borate will remain a complex multi-step process including the formation of NaBH_4_. Therefore, there are serious doubts that AB fuel cells would be more practical than the more simple borohydride fuel cells, which have a higher energy density [185].

#### Regenerative organic fuel cells

The direct use of organic hydrides in LFCs as virtual hydrogen carriers that generate stable organic molecules, protons and electrons upon reversible electrooxidation (Equation 6 direct and reverse) could provide an attractive alternative to hydrogen gas or metal hydride storage coupled with conventional hydrogen-air fuel cells [36–37]. The overall reaction is described by Equation 7.

This ‘virtual hydrogen’ scheme proposed by the Energy Frontier Research Center for Electrocatalysis, Transport Phenomena, and Materials, which was funded by DOE and is led by General Electrics (GE) avoids the release of hydrogen gas thus by-passing issues associated with hydrogen storage, transportation and safety. Compared to a hydrogen-on-demand design that includes thermal decomposition of organic hydrides in a catalytic reactor [38], this approach is much simpler, does not require additional dehydrogenation catalysts, heat exchangers and has higher energy density [36–37].

This concept of regenerative fuel cells (RFC) was demonstrated for vaporized organic fuels such as cyclohexane/benzene (OCV = 920 mV) [186] and isopropanol/acetone (OCV of the cell = 790 mV) [46] couples by using a Pt/C electrocatalyst. The power density was low (15 and 78 mW/cm^2^, respectively) but may be improved by Pt alloying, e.g., with Ni [47]. An attempt to use a neat liquid fuel (*N*-ethyldodecahydrocarbazole, dodecahydrofluorene) while using a PtRu catalyst resulted in a high OCV but very low current density [187]. Therefore, the development of effective and selective electrocatalysts for liquid organic fuels and compatible PEMs remains a major challenge.

A concept of a thermally regenerative fuel cell has been proposed by Ando et al. [188–189]. In this approach power is generated by electrochemical hydrogenation of acetone to IPA at the positive electrode and the dehydrogenation of IPA at the negative electrode by using low-grade heat. In another version, hydrogen that is generated through the thermal catalytic decomposition of IPA (Equation 27) serves as a proton source in the reactions in Equation 2 and Equation 13 (reverse) [188]. The OCV of cells based on the reaction in Equation 27 with Nafion® 117 PEM was close to theoretical but the voltage sharply decreased with the current density showing a peak power density of only about 650 µW/cm^2^ [188]. When IPA was used as hydrogen source, the cell power was very low (less than 20 mW). The cell efficiency peaked at IPA concentrations of 50–70%. The hydrogenation of acetone at the cathode was the rate-determining reaction. Replacing the PtRu/C electrocatalyst with PdRu/C or PdFe/C [190] or addition of sulfuric acid to the catholyte [191] increased the cell OCV by a factor of 2 to 4. The electrochemical hydrogenation of acetone dissolved in water and cyclohexane in a polymer electrolyte reactor showed that hydrogen evolution was a competing reaction with a similar reaction rate [192]. In a cell with a PtRu catalyst and a Nafion® 117 PEM, a maximum rate and current efficiency was achieved at an acetone concentration of about 3.5 M [193]. Increasing the cell temperature increases the reaction rate and current efficiency (up to about 60%) [193].

[27]
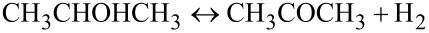


These reactions were successfully implemented in a vapor-fed IPA-based fuel cell with an air cathode [46,194], and they could be used potentially in a RLFC.

### Current trends and outlook

#### Fuel cell design

There are three major types of low temperature LFCs based on the type of ion exchange membrane: proton exchange (PEM), cation (alkali metal) exchange (CEM) and anion (hydroxyl) exchange (AEM). Recently, novel concepts of two layered (acidic–basic) [80] and three layered (basic–acidic–basic) [195] membranes have been proposed. PEM LFCs dominated in the literature but recently AEM LFCs got more attention [196]. In alkaline media the alcohol fuel oxidation rate is higher, and the overpotential for the ORR is lower [197]. In addition, OH^−^ ions and fuel molecules move in the opposite direction in an AEM, therefore potentially reducing the fuel crossover rate. However, these advantages are offset by the low conductivity of AEMs (at least an order of magnitude lower than that of PEMs) and by their lower stability [198]. Direct comparison of LFCs running on MeOH, EtOH and iPrOH in alkaline (0.5 M KOH) and acidic (0.1 M HClO_4_) solutions with Fumapem® FAA-2 (FumaTech) and Nafion® 115 (DuPont) membranes, respectively, while using the same PtRu catalyst showed that despite their higher current densities in alkaline solutions, the peak power density of the acidic cells was more than one order of magnitude higher than that of alkaline cells [199]. In a membrane-less fuel cell based on laminar flow, which is considered for small-scale portable applications, the OCV and current density was indeed higher in alkaline media (but still low compared with the AEM cell design) [200]. Noteworthy, for ethanol LFCs with an air cathode the highest power density was reached with an AEM [77]. Unfortunately, the conductivity of AEMs cannot be increased by operating at higher temperatures because of the low chemical stability of these membranes towards bases. Another disadvantage of alkaline LFC, especially targeting the full oxidation like DMFC, is the formation of alkali metal carbonates and bicarbonates that crystallize in the electrolyte-filled pores thus blocking the ion transport. However, only AEMs could be used with nitrogen and boron hydride fuels. DMFC with a sodium conducting CEM (Nafion®) have been proposed, but they showed very low power densities (9 mW/cm^2^) [201]. Recently, a cell using 2 M EtOH in 2 M KOH with a KOH-modified Nafion® 112 membrane and a PtRu anode catalyst showed a peak power density comparable with ethanol-powered LFCs with Nafion® PEM [69], PBI/KOH [78] and Tokuyama [77] AEMs. Even better performance was demonstrated for a LFC running on 3 M EtOH in 5 M NaOH with a NaOH-modified Nafion® 112 CEM, a PdNi/C anode catalyst and a FeCo HYPERMEC^TM^ cathode catalyst [202]. Compared to the analogous cell with a Tokuyama A201 AEM it showed a higher power density (135 vs 115 mW/cm^2^) and a stable discharge behavior at 90 °C [202]. This finding opens a possibility to run alkaline LFCs at higher temperatures. The formation of a base at the cathode of CEM LFCs is an issue that needs to be solved (for example by recycling the base to the anode).

Although oxygen is easily obtained from the atmosphere and there is no need for oxidant storage, sluggish kinetics of the ORR prompted search for a fuel cell cathode with better kinetics. Hydrogen peroxide cathodes allowed for the highest power densities for LFCs (vide supra). A bromine cathode has a much smaller overpotential compared to O_2_ and is used in hydrogen–bromine fuel cells [203]. Another liquid cathode comprising a water soluble oxidant, e.g., iron [204] or vanadium [205] complexes and a catalyst such as a polyoxometalate was proposed by ACAL Energy. The reduced catholyte is reoxidized in a separated regeneration unit by air oxygen, which serves as the ultimate oxidant.

#### Electrocatalysts

The anodic oxidation of fuels in LFCs remains the main challenge. Known anode electrocatalysts for LFCs are either too expensive or have low activity, and chemical and thermal stability, or are not selective enough. Pt is the most active electrocatalyst known for oxidation of organic fuels, but it is poisoned by reaction products. To overcome these shortcomings, several approaches are currently investigated, including decreasing the high loading of PGM metals (e.g., by increasing the dispersity or by the use of core–shell structures) or replacing platinum with less expensive PGMs (Pd) or base metals (in alkaline LFCs), while adjusting the electronic structure by adding adatoms, alloying, and using active or constraining supports.

In PEM LFCs the alloying of platinum with other metals, such as Sn, Ru, Ni, Co, etc. leads to more stable catalysts for alcohol oxidation with Pt–Sn alloys being the most active [206]. In alkaline media, an unsupported PdIn_3_ catalyst synthesized by the sacrificial support method had an increased surface area (40 m^2^/g) and demonstrated a very high activity in the oxidation of ethylene glycol and glycerol [207].

The anode catalyst support may play an important role. Pd nanoparticles supported on a Ni–Zn phase on carbon showed an excellent electrocatalytic activity in the oxidation of ethylene glycol and glycerol with peak current densites of 3300 and 2150 A/g Pd, respectively [208]. A Pd catalyst supported by multi-wall carbon nanotubes (MWCNT) showed superior performance compared to that on the conventional Vulcan XC-72 support, which was attributed to both a higher dispersion of Pd nanoparticles and to intrinsic properties of the support [114]. Nitrogen doping of porous carbon nanospheres increases the activity of Pt nanoparticles towards methanol electrooxidation [209]. Polystyrene spheres (diameter 700 nm) were used as a support for AuNi catalysts to form a three-dimensional core–shell structure with improved fuel diffusion into the catalyst layer, which showed a high activity in glycerol electrooxidation in alkaline medium [210]. A study of the electrooxidation of glycerol and EG on Au and Pt nanoparticles supported on different carbon surfaces suggested that oxygenated species formed on their surface serve as additional oxygen suppliers for the oxidation of intermediates adsorbed on the metal particles [211].

Oxidation of fuels on cathode catalysts and the resulting adsorption of intermediate products on the surface due to the crossover effect reduce their activity in the ORR by a factor of 3 to 7; this emphasizes the importance of the development of stable ORR electrocatalysts. It was found that some elements (Ru, Co, and Mo in acid media and Ag, Au in alkaline media) in binary and ternary compositions improve the resistance of the catalysts to poisoning [196].

#### Fuel development

Inorganic fuels such as hydrazine and sodium borohydrides are fully consumable as the intermediate products are oxidized more easily than the fuel. The only fully consumable organic fuel is methanol, and its disadvantages (high crossover rate leading to the use of diluted solutions, catalyst poisoning by reaction intermediates, toxicity, etc.) are well documented [48–55]. Among MeOH, EtOH and iPrOH fuels, the latter demonstrated the highest current and power densities on a PtRu electrocatalyst in both alkaline and acidic LFCs [199] with the exception of fuel cells with a PBI/PA membrane [71]. Contrary to alkaline LFCs, in acidic LFCs replacing methanol with C_2_-alcohols leads to a sharp decline of the power density [212]. The power densities in LFCs running on 1 M alcohol solutions with a PtRu anode catalyst follow a similar order: isopropanol > methanol > ethanol > *n*-propanol > *n*-butanol [73]. The electrooxidation rate of different alcohols on a Pd electrode decreased in the row *n*-propanol > isopropanol > ethanol > ethylene glycol > glycerol > methanol, while on a Pt electrode a different order was observed: isopropanol > ethanol > glycerol > ethylene glycol > *n*-propanol > methanol [213]. In LFCs with a Pd/MWCNT anode catalyst glycerol (5 wt %) delivered higher power density that was higher than that of 10 wt % EtOH but lower than that of 10 wt % MeOH [114].

For fuels containing C–C bonds, a complete electrochemical oxidation to CO_2_ and H_2_O seems to be unachievable at practical current densities, at least at the operational temperatures of conventional PEM fuel cells and with the known electrocatalysts. Another approach is a partial electrochemical oxidation of the fuel to compounds that are stable under the working conditions of the cell. For example, ethanol in alkaline media can be selectively converted to acetic acid, which is isolable as alkali metal acetate [114], isopropanol to acetone [88], and cyclohexane to benzene [186]. Clearly, a partial oxidation yields a lesser system energy density compared to complete oxidation. Nevertheless, in many cases the energy density is still much higher than that of conventional batteries. The use of polyhydric alcohols such as diols may substantially increase the energy density compared with monohydric alcohols [214]. Thermodynamic analysis and DFT computation show that the most energy-dense fuels for RLFCs are acyclic compounds and nitrogen-containing saturated heterocycles, especially those with five-membered rings [39]. Electrochemical oxidation of the former is very difficult and was only done in the vapor phase. The latter can be electrochemically oxidized by using inexpensive Ni catalysts [215]. However, they are incompatible with highly acidic PEMs, and the electrochemical oxidation of these compounds in alkaline media leads to the formation of oxygenated species [216]. Polyhydric alcohols having a lower energy density but being compatible with both PEMs and AEMs seem to be a reasonable compromise.

The electrochemical hydrogenation of carbonyl compounds and organic acids on Pt, Pd and Raney Ni electrocatalysts is well known [217] and it can be potentially used in RLFCs. The main challenge is the low selectivity due to the competing hydrogen evolution reaction. If the electrochemical hydrogenation was too slow or uneconomical (e.g., poor efficiency), the dehydrogenated or oxidized fuel could be regenerated ex situ through the well-known catalytic hydrogenation with molecular hydrogen in the gaseous or liquid phase. These hydrogenation processes are well developed for the hydrogenation of aromatic compounds to the related cyclic aliphatic compounds, acetic acid to ethanol, diketones to diols, etc. In this scheme the spent fuel would be collected at refueling stations, sent to a centralized plant for regeneration, and shipped back to the refueling stations in its hydrogenated form by using the existing infrastructure.

## Conclusion

The development of cost-competitive LFCs would eliminate a major hurdle in the broad implementation of hydrogen fuel cells: the high cost of transportation and the absence of an infrastructure for hydrogen delivery. In the case of an implementation of LFCs, the existing liquid fuel infrastructure could be used. So far, the most developed organically fueled LFCs, DMFCs, have only reached power densities lower (by large factors) than those achievable by hydrogen fuel cells, even when using much higher Pt loadings [48–56]. Only LFCs using expensive (NaBH_4_) or toxic (N_2_H_4_) fuels exhibit power densities comparable with hydrogen powered fuel cells. Therefore, the development of highly active and robust electrocatalysts is critical.

RLFCs based on electrochemical dehydrogenation/hydrogenation have a lower energy density compared with cells based on the complete oxidation of fuels, but they are very attractive for energy storage applications. A variety of organic fuels with tunable properties can be used, but the development of catalysts capable to selectively catalyze electrochemical dehydrogenation and hydrogenation reactions, as well as compatible ion-exchange membranes, is necessary. Fuels forming aromatic structures or carbonyl bonds through the extraction of hydrogen from the C–H or the O–H bonds, respectively, may provide much higher energy densities. Currently, oxygenated fuels seem to be the best compromise between energy density, easiness of electrooxidation and compatibility with existing acidic membranes. It is also possible that basic nitrogen-containing heterocyclic compounds that have good thermodynamics and high energy densities could be used with basic membranes.

Research efforts should be focused on development of inexpensive, selective and active electrocatalysts and minimizing the fuel crossover in ion-conducting membranes. Increasing of the LFC working temperature above 150 °C may ease the requirements for the electrocatalyst by increasing the fuel electrooxidation rate, while reducing electrocatalyst poisoning by intermediate products. For that purpose the development of new ion-conducting membranes that have a high conductivity at a low relative humidity is necessary.
